# Knockdown of RIPK2 Inhibits Proliferation and Migration, and Induces Apoptosis *via* the NF-κB Signaling Pathway in Gastric Cancer

**DOI:** 10.3389/fgene.2021.627464

**Published:** 2021-02-09

**Authors:** Qian Yang, Shan Tian, Zhengru Liu, Weiguo Dong

**Affiliations:** ^1^Department of Gastroenterology, Renmin Hospital of Wuhan University, Wuhan, China; ^2^Key Laboratory of Hubei Province for Digestive System Disease, Wuhan, China; ^3^Central Laboratory, Renmin Hospital of Wuhan University, Wuhan, China

**Keywords:** RIPK2, gastric cancer, proliferation, migration, apoptosis, NF-κB signaling

## Abstract

RIPK2 is a 62 kDa protein and a member of the receptor interacting protein kinases (RIPK) family. It was previously demonstrated that RIPK2 might play a role in promoting malignant tumor progression; however, the precise function of RIPK2 in the onset and progression of gastric cancer (GC) remains unclear. In the current study, we investigated the role of RIPK2 in GC. First, we explored the expression levels of RIPK2 in multiple cancers, including GC, using a bioinformatics approach. We constructed the RIPK2-associated protein-protein interaction network using the search tool for the retrieval of interacting genes/proteins for gene ontology and Kyoto encyclopedia of genes and genomes analysis. Next, we compared the RIPK2 expression levels between GC cells and normal gastric mucosal epithelial cell (GES-1) using reverse transcription quantitative PCR analysis. We downregulated the expression of RIPK2 in GC cells to determine the effects of RIPK2 on cell growth, migration, and apoptosis. Finally, we used western blotting to investigate the RIPK2 downstream signaling pathway involved in the regulation of GC progression. Our results showed that RIPK2 was overexpressed in various tumor tissues, including GC, compared to non-cancer tissues. Moreover, RIPK2 expression was significantly upregulated in all four GC cell lines (MGC-803,SGC-7901, HGC-27 and AGS) comparing the GES-1 cells. Silencing of RIPK2 suppressed GC cell growth by inhibiting migration, and inducing apoptosis through the nuclear factor-κB (NF-κB) signaling pathway. In summary, we demonstrate that RIPK2 plays an important role in modulating GC cell proliferation, migration, and apoptosis through the NF-κB signaling pathway. Therefore, RIPK2 functions as a potential oncogene. We believe that RIPK2 can be used as a candidate biomarker, as well as a diagnostic tool, and the therapeutic target for GC.

## Introduction

Gastric cancer (GC), the third most common malignancy, is a primary cause of tumor-related deaths worldwide (Bray et al., 2018). The existing first-line therapeutic approaches for GC, such as surgery and chemotherapy, are still limited in their ability to improve the prognosis. Early detection and treatment for GC may reduce pain and have a vital impact on the potential for complete recovery. But unfortunately, majority of GC patients often diagnosed at Stage II or III (Choi et al., 2015, 2019). The pathogenesis of GC is diverse, involving multiple genes and cellular pathways. Despite numerous efforts to study the underlying mechanisms of development in GC, many details regarding these aspects remain unclear. So far, esophagogastroduodenoscopy is a sensitive clinical screening method for GC; however, due to the invasiveness of the examination, poor patient compliance, and psychological fear, its application is underutilized. Currently, targeted cancer therapies serve as a critical cornerstone of precision medicine. Thus, development of the targeted therapy has required the identification of good targets, and these specific molecular targets should play a critical role in regulating cancer cell growth, apoptosis, and survival. On the other hand, biochemical markers could be used as non-invasive diagnostic tools; therefore, it is imperative to investigate novel potential molecular markers for the diagnosis and treatment of GC.

Receptor interacting serine/threonine kinase 2 (RIPK2, also known as RIP2, RICK, and CARD3), which is known for its role in immunity and inflammation, is a potent activator of nuclear factor κB (NF-κB) (McCarthy et al., 1998; Park et al., 2007). Activation of RIPK2 may subsequently activate IκB kinases (IKKs), followed by the translocation of the NF-κB dimers (e.g., p65/p50) into the nucleus to trigger the transcription of target genes (Hoesel and Schmid, 2013). Previous studies have investigated the importance of RIPK2 in inflammatory pathogenesis and (auto) immune diseases, such as multiple sclerosis (Shaw et al., 2011), pancreatitis (Duggan et al., 2017) and inflammatory bowel disease (Chen et al., 2019). Therefore, RIPK2 might play a vital role in overactive inflammatory responses observed in various diseases, including cancer. Our previous study demonstrated that RIPK2 was overexpressed in colorectal cancer (CRC), and, in combination with *Fusobacterium nucleatum*, was involved in the regulation of CRC metastases (Chen et al., 2020). In addition, RIPK2 played a crucial role in the formation and progression of oral squamous cell cancer (Wang et al., 2014), inflammatory breast cancer (Zare et al., 2018). Nevertheless, the role of RIPK2 in GC remains unclear.

In the current study, we demonstrated that RIPK2 was overexpressed both at mRNA and protein levels in GC tissues. In addition, we further investigated the biological effects of RIPK2 on GC cell proliferation, apoptosis and migration. These findings indicate that RIPK2 plays a vital role in GC cell tumorigenicity and proliferation by regulating NF-κB signaling and suggest that RIPK2 could be a potential target for GC therapy.

## Materials and Methods

### RIPK2 Expression in Human Cancers Using Pan-Cancer Analysis

RIPK2 mRNA expression levels in various solid tumors were identified using the oncomine (Rhodes et al., 2004)^1^ and tumor immune estimation resource (TIMER) (Li et al., 2017)^2^ databases. In TIMER, we used the DiffExp module to analyze the differential expression between tumor and adjacent normal tissues for RIPK2 across all the cancer genome atlas (TCGA)^3^.

### RIPK2 Expression in Gastric Cancer

To evaluate the expression of RIPK2 in GC, we used three independent databases: TCGA (April, 2020), national center for biotechnology information- (NCBI) gene expression omnibus (GEO) (Barrett et al., 2013)^4^ [GSE19826 (Wang et al., 2012), GSE79973 (Jin et al., 2017), and GSE33335 (Cheng et al., 2013)], and oncomine. We used receiver operating characteristic (ROC) curves to assess the diagnostic value of RIPK2. The protein expression level of RIPK2 was investigated using the human protein atlas (Uhlen et al., 2017) (HPA)^5^ database.

### Identification of RIPK2-Associated Proteins and Functional Enrichment Analysis

The construction of the RIPK2-associated protein network was carried out using search tool for the retrieval of interacting genes/proteins (STRING) (Szklarczyk et al., 2015)^6^ (interaction score > 0.9); stringApp (a plugin of Cytoscape, v3.7.2) software was used to visualize the network. The gene ontology (GO) (Ashburner et al., 2000) and Kyoto encyclopedia of genes and genomes (KEGG) (Kanehisa, 2002) analyses for RIPK2-associated proteins were applied using the database for annotation, visualization and integrated discovery (DAVID) (Huang et al., 2007)^7^; *P* < 0.05 was identified as a significance threshold. The molecular functions of the genes were visualized using Sangerbox^8^.

### Validation Based on Human GC Samples

To further verify the data from GEO, TCGA, and oncomine, we detected the RIPK2 mRNA expression using quantitative reverse transcription- (qRT) PCR. Human GC samples (13 GC tissues and 13 matched non-tumor tissues) were obtained from the Renmin Hospital of Wuhan University (Wuhan, China). Informed consent was provided by all participants, and the research protocol was approved by the Institutional Review Board of the Renmin Hospital of the Wuhan University.

### Cell Culture

All cell lines were obtained from the Key Laboratory of Hubei Province for Digestive System Disease (Wuhan, Hubei, China). GES-1 (gastric mucosal epithelial cell line) and GC cell lines SGC-7901, MGC-803, AGS, and HGC-27 were cultured in DMEM/F-12 medium (SH30023; HyClone, United States) containing 10% fetal bovine serum (FBS, sijiqing, Hangzhou, China) at 37°C in a humidified atmosphere of 5% CO_2_.

### Cell Transfection

Cells were plated overnight to grow to 40–50% confluence at the time of transfection. siRNAs targeting the human RIPK2 gene (siRNA-RIPK2) and non-targeting negative control siRNA (siRNA-NC) were purchased from Suzhou GenePharma Co., Ltd. (Shanghai, China). The siRNAs were as follows: si-RNA-RIPK2: sense, 5′-CAAUAUGACUCCUCCUUUATT-3′, antisense, 5′-UAAAGGAGGAGUCAUAUUGTT-3′; non-targeting siRNA (siRNA-NC): sense, 5′-UUCUCCGAACGUACGUTT-3′, antisense, 5′-ACGUGACACGUUCGGAGAATT-3′. Lipid-based transient transfections were performed using Lipofectamine 6000 Transfection Reagent (Biyuntian, Shanghai, China) according to the manufacturer’s instructions. Six hours later, the culture medium was replaced with fresh medium containing 10% FBS. After 48–72 h of transfection, fluorescence, quantitative reverse transcription- (qRT) PCR and western blotting were used to assess the transfection efficiency.

### RT-qPCR

Total RNA was extracted using Trizol reagent (15596-026; Invitrogen, Carlsbad, CA, United States) and then reverse-transcribed complementary DNA (cDNA) was synthesized using a PrimeScript^TM^RT reagent Kit with gDNA Eraser (RR047A; Takara, Shiga, Japan), following the manufacturer’s instructions. The conditions of RT-PCR were 37°C for 15 min, 85°C for 5 s, and held at 4°C. Then, the cDNA was diluted four times with nuclease-free water, and the RIPK2 mRNA expression (sense: 5′- GAATCATGTGGATCCTCTCAGC-3′; anti-sense: 5′-TGATTTCCAGGACAGTGATGC-3′) was detected by real-time PCR using SYBR ^®^ Premix Ex Taq^TM^ (RR420A, Takara). The reaction conditions were as follows: 95°C for 30 s, followed by 40 cycles at 95°C for 5 s, and 60°C for 30 s in 7900 real-time PCR system (Applied Biosystems, CA, United States). GAPDH (sense: 5′-CATCATCCCTGCCTCTACTGG-3′; anti-sense: 5′-GTGGGTGTCGCTGTTGAAGTC-3′) was used as the internal control. The relative RIPK2 mRNA expression was calculated using the 2^−ΔΔCT^ method.

### Western Blotting

Total proteins were extracted from transfected AGS and HGC-27 cells. Western blotting was performed according to standard protocols and as previously described (Li et al., 2019). Cell lysates were separated by 10–12% sodium dodecyl sulfate- polyacrylamide gel electrophoresis (SDS- PAGE) and transferred to a nitrocellulose membrane. After blocking with 5% non-fat milk for 1 h, the membranes were incubated with primary antibodies at 4°C overnight. Primary antibodies used were as follows: anti-RIPK2 (1:1,000, 15366-1-AP; Proteintech, Wuhan, China), anti-Cleaved-caspase 3 (1:1,000, #9661S; CST, United States), anti-Bcl-2 (1:1,000, #2872T; CST, United States), anti-Bax (1:1,000, 50599-2-Ig; Proteintech, Wuhan, China), anti-NF-κB P65 (1:1,000, #8242T; CST, United States), anti-p-NF-κB P65 (Ser536) (1:1,000, #3033; CST, United States), anti-IκBα (1:1,000, #4814T; CST, United States), anti-p-IκBα (Ser32) (1:1,000, #2859; CST, United States). Anti-GAPDH (1:10,000; AP0063; Bioworld, Nanjing, China) antibody was used as a loading control. Then, the membranes were washed three times with Tris-buffered saline-tween 20 (TBST), and incubated with species-specific secondary antibodies for 1.5 h at room temperature. After washing with TBST three times, the membranes were then detected with enhanced chemiluminescence reagent (Servicebio Technology Co., Wuhan, Chnia) and were observed by the Bio- Rad ChemiDoc ^®^ Touch Imaging System (BioRad, Hercules, United States). The image J software was used to analyze the results, and all protein expression levels were assessed relative to GAPDH expression.

### Cell Viability Assay

HGC-27 and AGS cells (2,000 cells per well) were seeded into 96-well plates and transfected with siRNA-RIPK2 and siRNA-NC. Cell Counting Kit-8 (CCK-8, Biyuntian, Shanghai, China) reagent 10μl was added to each experimental well after 0, 24, 48, and 72 h, respectively. Cell proliferation rates were measured using a microplate reader (BD Biosciences, United States) at 450 nm.

### Clone Formation Assay

Twenty-four hours after AGS and HGC-27 cells were transfected with siRNA-RIPK2 or siRNA-NC, infected cells (300 cells per well) were incubated in six-well plates. The transfected cells were cultured at 37°C for 13 days. Then, the cells were fixed with methyl alcohol for 10 min and stained with 4% crystal violet solution. The colonies in each group were washed before counting. ImageJ software (ImageJ 1.52a, United States) was used to analyze and quantify the number of colonies.

### Analysis of Apoptosis

AGS and HGC-27 cells were transfected as described in section “Cell Transfection” and cultured for another 24 h to perform the apoptosis analysis. The cells were harvested and washed with ice-cold PBS for twice, and then the cell concentration was adjusted to 1 × 10^6^ cells/ml. Next, cells were stained with 5 μl Annexin V-FITC (Biyuntian, Shanghai, China) for 10 min. And, the cells were centrifuged at 1,000 × g for 5 min to remove the supernatant, and then suspended in binding buffer. Finally, the cells were stained with 10 μl propidium iodide (Biyuntian, Shanghai, China) for 5 min in the dark and analyzed by flow cytometry (BD Biosciences) to evaluate the rate of apoptosis.

### Wound-Healing Assay

Cells were transfected and cultured to 90% confluence in a six-well plate. Then, wounds were scratched with a sterile pipette tip and cells were incubated in the medium without FBS. Distance migrated by the cells was recorded at 0 and 24 h after wound scratching. Microscopy images detected cells that migrated into the wound area. Three independent experiments were performed.

### Cell Migration Assays

To assess cell migration, the transwell migration assay was performed. Transfected cells were seeded in the top chambers containing 1% FBS, while medium containing 20% FBS was added to the lower chambers. After 24 h, the migrated cells on the bottom part of the membrane were fixed with 70% methanol and stained with 0.1% crystal violet. The cells from the top of the membrane were carefully removed, while the migrated cells were quantified in three random microscopic fields.

### Statistical Analysis

Statistical analyses were carried out using R version 3.6.1 and GraphPad Prism7 (GraphPad software, Inc., San Diego, CA, United States). All experiments were repeated at least thrice. The data are presented as mean ± SEM. Data were analyzed using either a two-tailed Student’s *t*-test, or a two-way ANOVA with Sidak’s multiple comparisons test. Statistical significance was set at *P* < 0.05 (^∗^*P* < 0.05, ^∗∗^*P* < 0.01, ^∗∗∗^*P* < 0.001, ^****^P < 0.0001).

## Results

### mRNA Expression Levels of RIPK2 in Pan-Cancer

**FIGURE 1:**
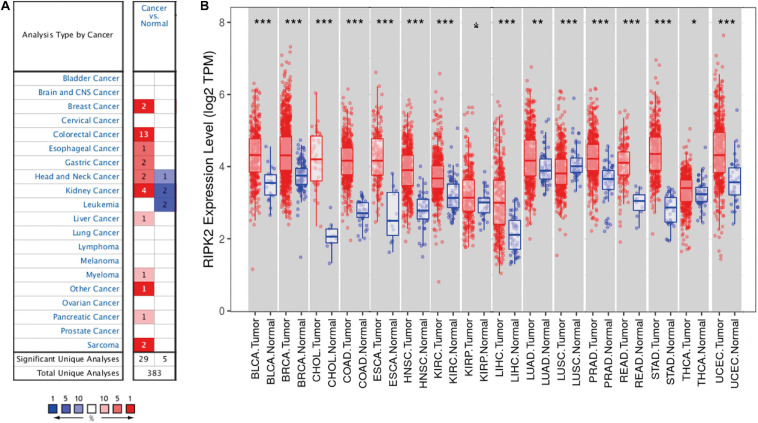
RIPK2 expression levels in cancers. **(A)** Differential expression of RIPK2 in tumor tissues compared with normal tissues in oncomine. Number in each cell indicates the number of datasets; red indicates that RIPK2 is upregulated in corresponding cancer, blue indicates that RIPK2 is downregulated in corresponding cancer. **(B)** Human RIPK2 expression levels in different types of tumors from TCGA data in TIMER. *P < 0.05, **P < 0.01, ***P < 0.001. TCGA, the cancer genome atlas; TIMER, tumor immune estimation resource.

To determine the differences in RIPK2 mRNA expression in tumor and adjacent normal tissues, RIPK2 expression levels were analyzed using the oncomine database over a cancer-wide range. This analysis showed that the RIPK2 had a higher expression in gastric, breast, esophageal, colorectal, head and neck, liver, kidney, as well as pancreatic cancers, myeloma and sarcoma, compared to that in the respective normal tissues (Figure 1A). In addition, RIPK2 expression was low in head and neck, and kidney cancers, and leukemia in some datasets. Supplementary Table 1 summarizes the details of RIPK2 expression in different types of cancers.

To verify the expression level of RIPK2 in human cancers, we analyzed RIPK2 expression based on TCGA data by TIMER. As shown in Figure 1B, RIPK2 was differentially expressed in tumor tissues compared to that in the adjacent normal tissues. RIPK2 was significantly upregulated in BLCA (bladder urothelial carcinoma), BRCA (breast invasive carcinoma), ESCA (esophageal carcinoma), COAD (colon adenocarcinoma), CHOL (cholangiocarcinoma), HNSC (head and neck cancer), STAD (stomach adenocarcinoma), READ (rectum adenocarcinoma), KIRC (kidney renal clear cell carcinoma), LUAD (lung adenocarcinoma), UCEC (uterine corpus endometrial carcinoma), THCA (thyroid carcinoma), LIHC (liver hepatocellular carcinoma), and PRAD (prostate adenocarcinoma) compared to that in their respective normal tissues. Meanwhile, RIPK2 expression was significantly lower in LUSC (lung squamous cell carcinoma) compared to that in the normal tissues.

### RIPK2 Was Consistently Upregulated in GC

**FIGURE 2:**
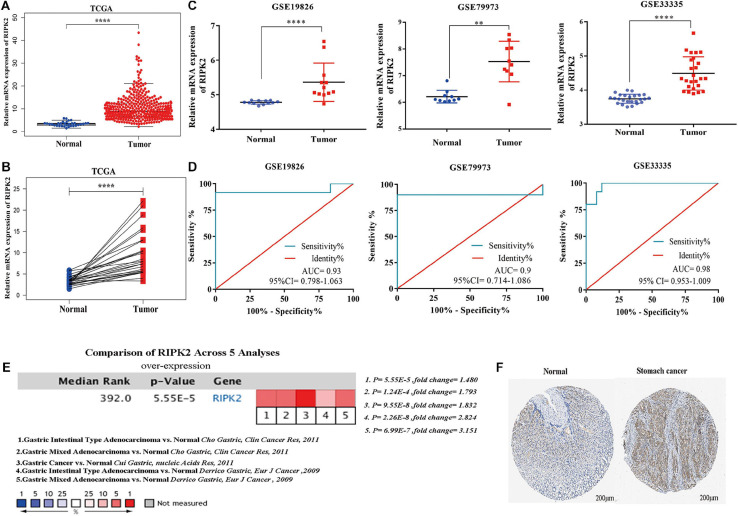
RIPK2 expression in gastric cancer. **(A,B)** Expression level of RIPK2 in TCGA gastric tumor and normal tissues (**A:** Tumor = 375, Normal = 32; **B:** Tumor = 28, Paired non-tumor tissues = 28; ^****^P < 0.0001; two-tailed *t*-test). **(C)** RIPK2 expression was upregulated in gastric cancer tissues compared to that in the normal gastric tissues; based on the GSE19826 (^****^P < 0.0001, two-tailed *t*-test), GSE79973 (^∗∗^*P* < 0.001, two-tailed *t*-test) and GSE33335 (^****^P < 0.0001, two-tailed *t*-test). **(D)** ROC curve analysis of the RIPK2 mRNA expression levels in the GC datasets from the GEO database: GSE19826 (AUC = 0.93), GSE79973 (AUC = 0.9), GSE33335 (AUC = 0.98). **(E)** Oncomine database was applied to verify the increased expression of RIPK2 in various subtypes of gastric cancer tissues. **(F)** RIPK2 protein expression was higher in tumor tissues compared to normal tissues based on HPA database (Normal: id 2,471; male; 65 years old; staining negative; tumor: id 2,473; male; 59 years old; staining: moderate; intensity: medium; quantity: 75%; location: cytoplasm/membrane). ROC, receiver operating characteristic; AUC, area under the ROC curve; HPA, human protein atlas; GC, gastric cancer.

The mRNA expression of RIPK2 was highly overexpressed in GC tissues compared to that in the normal gastric tissues based on TCGA-stomach tumor database (Figures 2A,B) (A: 375 GC tissues, 32 normal gastric tissues; B: 28 GC tissues and paired non-tumor tissues) and GEO (Figure 2C) (GSE19826: 12 GC tissues, 12 matched non-cancer gastric tissues; GSE79973: 10 GC tissues and paired adjacent tissues; GSE 33335: 25 GC and 25 normal gastric tissues). In addition, the RIPK2 diagnostic value for GC was determined using the ROC curve analysis. The results of the ROC analysis were as follows: GSE19826 (area under the ROC curve (AUC) = 0.930, 95% confidence interval (CI): 0.798–1.063, *p* = 0.0003); GSE79973 (AUC = 0.9, 95% CI: 0.7141–1.086, *p* = 0.0025); GSE33335 (AUC = 0.98, 95% CI: 0.953–1.009, *p* < 0.0001) (Figure 2D). Moreover, RIPK2 also showed higher mRNA expression in GC tissues in the oncomine database (median rank = 392.0, *p* = 5.55E-5) (Figure 2E), and RIPK2 protein expression in GC was also increased compared to that in normal gastric tissues (Figure 2F).

### GO and KEGG Analysis of RIPK2-Related Proteins

**FIGURE 3:**
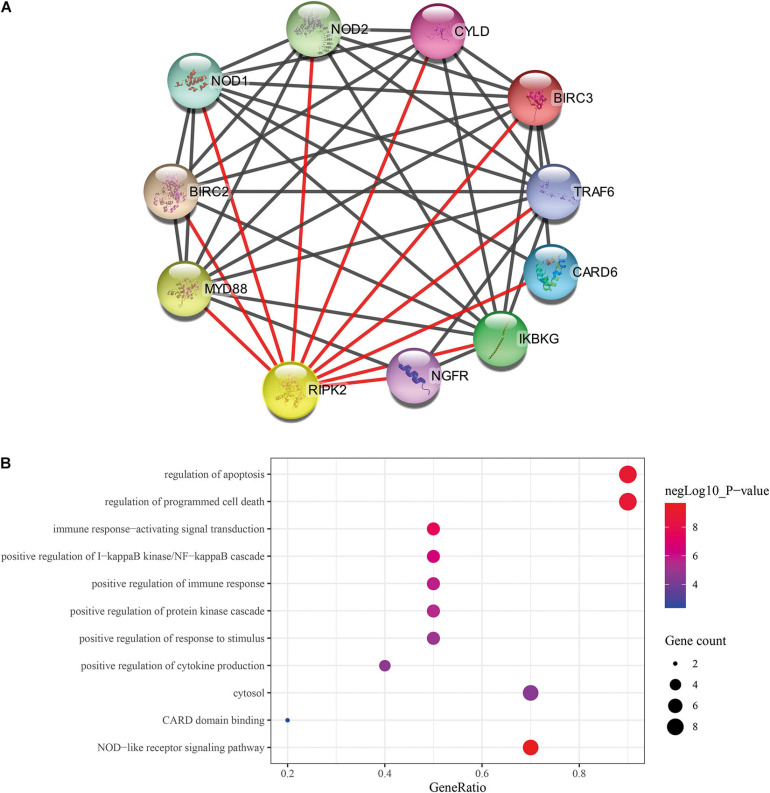
Identification of predicted interacting proteins of RIPK2 and the molecular function analysis. **(A)** The PPI network that was closely associated with RIPK2. **(B)** GO and KEGG analysis of the predicted proteins interacting with RIPK2. BP, biological process; CC, cellular component; MF, molecular function; PPI, protein–protein interaction; GO, gene ontology; KEGG: Kyoto encyclopedia of genes and genomes.

Previous studies showed that RIPK2 plays a role in tumor progression; however, these observations were limited to carcinomas of the breast (Zare et al., 2018), bladder (Zhang and Chin, 2014) and colon (Chen et al., 2020). To date, there is no clear evidence of the role of RIPK2 in GC. Thus, cytoscape stringApp was used to construct the protein-protein interaction (PPI) network of RIPK2 and its protein partners. Next, a network of proteins that were tightly associated with RIPK2 was established (Figure 3A). There were 11 nodes and 43 edges (mean node degree is 7.82, mean local clustering coefficient is 0.908, and PPI enrichment *p*-value is 9.25E-13) in this network. The 10 predicted RIPK2-interacting proteins included NOD1 (nucleotide-binding oligomerization domain containing 1), NOD2, CYLD (CYLD lysine 63 deubiquitinase), BIRC3 (baculoviral IAP repeat containing 3), TRAF6 (TNF receptor associated factor 6), CARD6 (caspase recruitment domain family member 6), IKBKG (inhibitor of nuclear factor kappa B kinase regulatory subunit), NGFR (nerve growth factor receptor), MYD88 (MYD88 innate immune signal transduction adaptor), and BIRC2 (baculoviral IAP repeat containing 2). The analysis of these genes will be done in the future. To investigate the pathway that might be involved in RIPK2-mediated gastric cancer progression, GO and KEGG pathway analyses were performed. GO analysis of these proteins in the PPI network showed that RIPK2 was closely related to the regulation of apoptosis, production of cytokines, and immune response. The KEGG pathway analysis indicated that RIPK2 was associated with NOD-like receptor and IκB/NF-κB signaling pathway (Figure 3B).

### Inhibition of RIPK2 Suppressed GC Cell Proliferation

**FIGURE 4:**
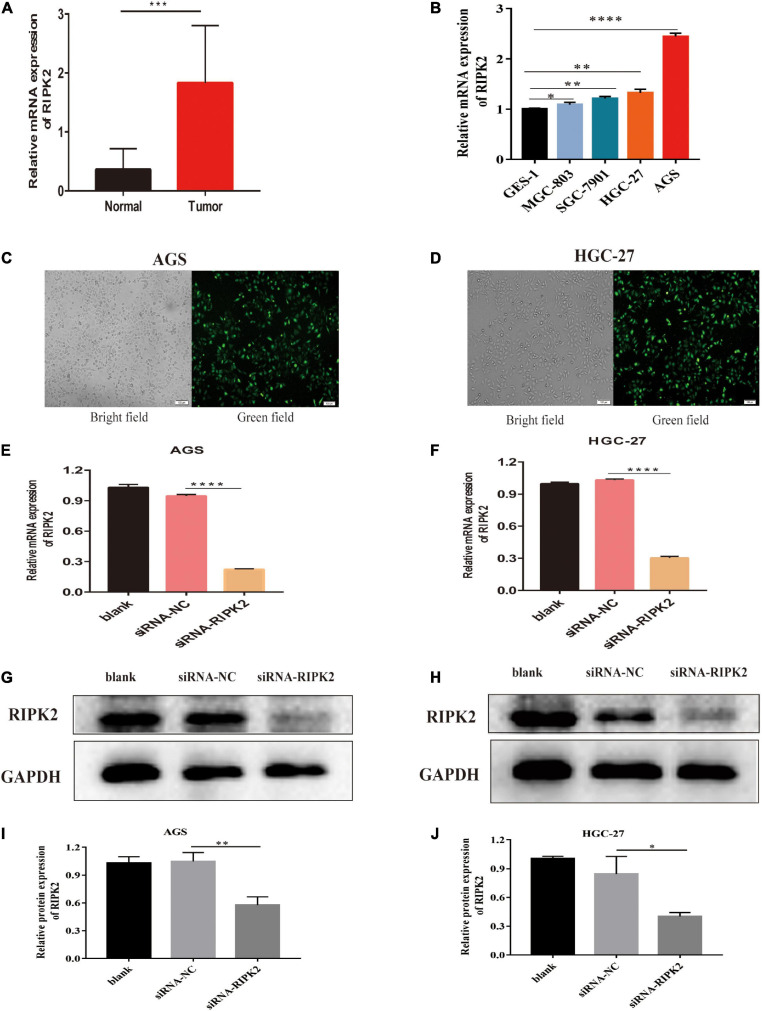
RIPK2 expression in gastric cancer tissues and cells. **(A)** The mRNA expression levels of RIPK2 in GC tissues (^∗∗∗^*P* < 0.001, versus normal gastric tissues; two-tailed *t*-test). **(B)** The mRNA expression levels of RIPK2 in GES-1 and GC cells (^∗^*P* < 0.5, ^∗∗^*P* < 0.01, ^****^P < 0.0001 versus GES-1; two-tailed *t*-test). Analysis of the transfected efficacy of RIPK2 knockdown in AGS and HGC-27 cells: **(C,D)** Bright-field images and the corresponding fluorescence images of transfected AGS and HGC-27 cells. **(E–J)** Expression levels of RIPK2 were examined after siRNA-RIPK2 transfection in AGS and HGC-27 cells by qRT-PCR and Western blotting (^∗^*P* < 0.05, ^∗∗^*P* < 0.01, ^****^P < 0.0001 versus siRNA-NC; two-tailed *t*-test). NC, negative control; GC, gastric cancer.

**FIGURE 5:**
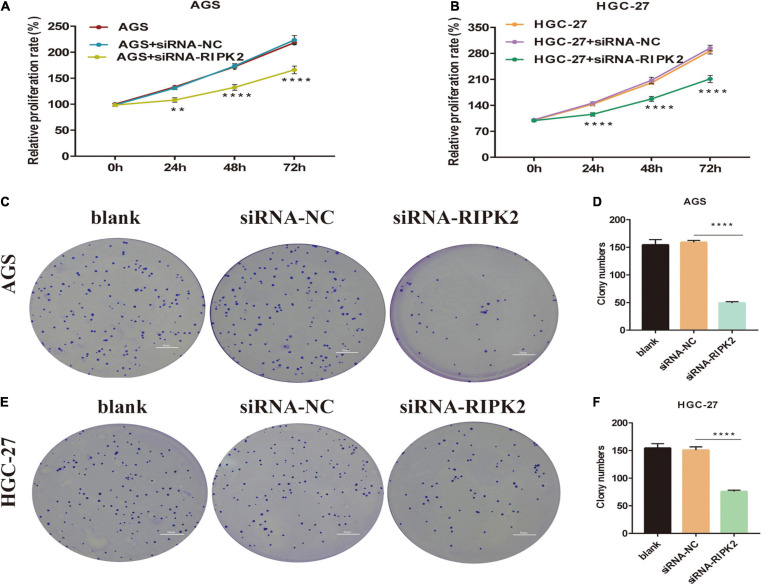
Knockdown of RIPK2 inhibited GC cell proliferation. **(A,B)** Cell counting kit-8 (CCK8) assays shows that downregulation of RIPK2 suppresses growth of AGS and HGC-27 cells (**P < 0.01, ****P < 0.0001 versus siRNA-NC; two-way ANOVA). **(C–F)** In the colony formation assay, silencing of RIPK2 decreases the colony number in AGS and HGC-27 cells (****P < 0.0001 versus siRNA-NC; two-tailed *t*-test). NC, negative control; GC, gastric cancer.

To determine the molecular function of RIPK2 in GC, we examined RIPK2 mRNA expression levels in GC tissues and four GC cell lines. As expected, RIPK2 was significantly overexpressed in 13 GC tissues (Figure 4A). And, RIPK2 expression was upregulated in MGC-803, SGC-7901, HGC-27, and AGS cells, compared to that in GES-1 cells (Figure 4B). Therefore, AGS and HGC-27 cells were selected for further analysis. HGC-27 and AGS cells were transfected with siRNA-RIPK2 and the non-targeting siRNA (siRNA-NC). Fluorescence, western blotting and qRT-PCR assays were used to determine the transfection efficiency of these two cell lines (Figures 4C–J). To investigate the effect of RIPK2 downregulation on GC cell growth, AGS and HGC-27 cells were transfected with siRNA-RIPK2, and cell proliferation was measured using the CCK8 assay. Our results demonstrated that the downregulation of RIPK2 significantly inhibited the proliferation of AGS and HGC-27 cells (Figures 5A,B). The knockdown of RIPK2 also significantly decreased the number of colonies compared to that in siRNA-NC transfected cells, as determined by the colony formation assay (Figures 5C–F). CCK8 cell proliferation and colony formation assays both showed that decreased expression of RIPK2 suppressed the proliferation of GC cells.

### Inhibition of RIPK2 Induced Apoptosis of GC Cells

**FIGURE 6:**
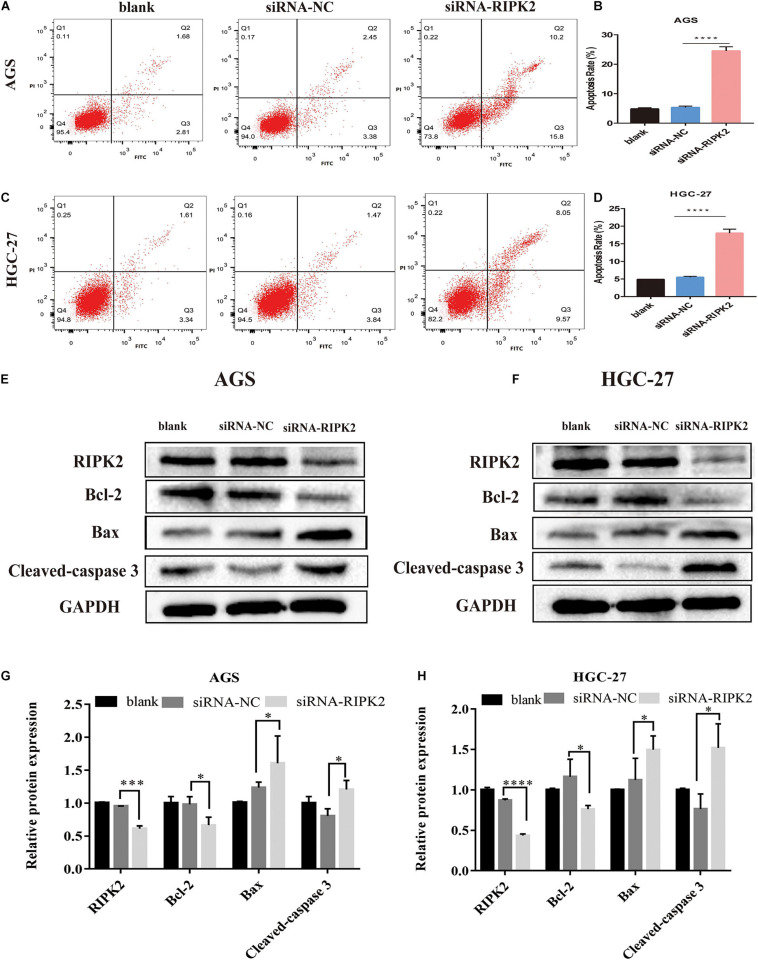
Knockdown of RIPK2 induced apoptosis. **(A–D)** Apoptotic rates were measured by Annexin V/PI staining and analyzed by an image flow assay in AGS and HGC-27 cells (****P < 0.0001 versus siRNA-NC; two-tailed *t*-test). **(E–H)** Western blot analysis of Bcl2, Bax and cleaved-caspase 3 protein levels in AGS and HGC-27 cells with downregulation of RIPK2 (*P < 0.05, ***P < 0.001, ****P < 0.0001 versus siRNA-NC; two-tailed *t*-test). AV, annexin V FITC; PI, propidium iodide; siRNA-NC, NC, negative control; GC, gastric cancer.

This suppression of cell proliferation prompted us to investigate the effect of RIPK2 silencing on apoptosis. Apoptosis of GC cells was evaluated using annexin V-FITC/PI flow cytometry. Silencing of RIPK2 increased the rate of apoptotic cells in AGS and HGC-27 cell lines compared to that in the control (siRNA-NC) (Figures 6A–D). In addition, western blotting analysis was showed that anti-apoptotic protein Bcl2 was decreased in both RIPK2-silenced AGS and HGC-27 cells, while the expression of apoptotic proteins Bax and cleaved caspase-3 were increased (Figures 6E–H). These data showed that the downregulation of RIPK2 promoted apoptosis in GC cells.

### Inhibition of RIPK2 Decreases the Migration Capacity of GC Cells

**FIGURE 7:**
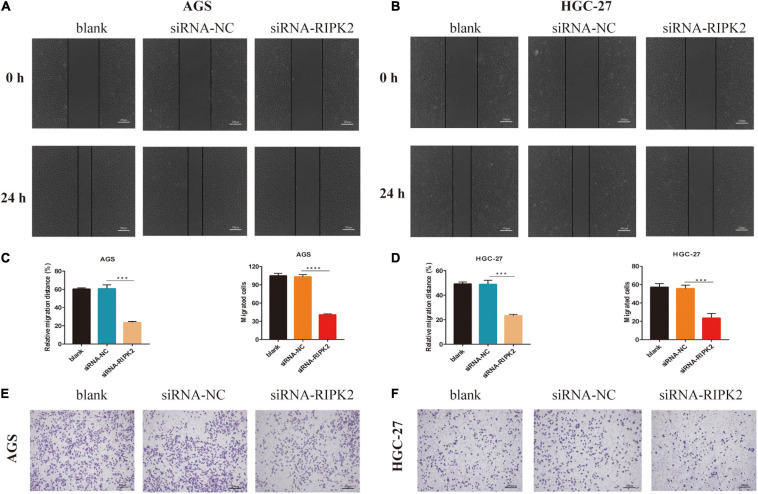
Knockdown of RIPK2 suppressed GC cell migration. **(A,B)** Representative images or wound healing assay, AGS and HGC-27 at 0 and 24 h, respectively (***P < 0.01 versus siRNA-NC; two-tailed *t*-test). **(C,D left)** Relative migration distance was analyzed in AGS and HGC-27 cells, respectively. **(C,D right)** The number of migrated cells was analyzed in AGS and HGC-27 cells, respectively. **(E,F)** Representative images of the migrated AGS and HGC-27 cells transfected with siRNA-RIPK2 or siRNA-NC (***P < 0.01, ****P < 0.0001 versus siRNA-NC; two-tailed *t*-test). GC, gastric cancer; NC, negative control.

To investigate whether RIPK2 silencing affected GC cell migration, we performed wound healing and transwell assays. It was shown that the wound closure of siRNA-RIPK2-transfected cells was suppressed compared to that in both AGS (Figures 7A,C-left) and HGC-27 (Figures 7B,D-left) control cells. Furthermore, the transwell migration assay indicated that the number of migrated AGS (Figures 7C-right, E) and HGC-27 cells (Figures 7D-right, F) was significantly reduced in siRNA-RIPK2 compared to that in the siRNA-NC group. These results suggested that RIPK2 plays a role in cell migration in GC cells.

### NF-κB Signaling Is Regulated by RIPK2 in GC

**FIGURE 8:**
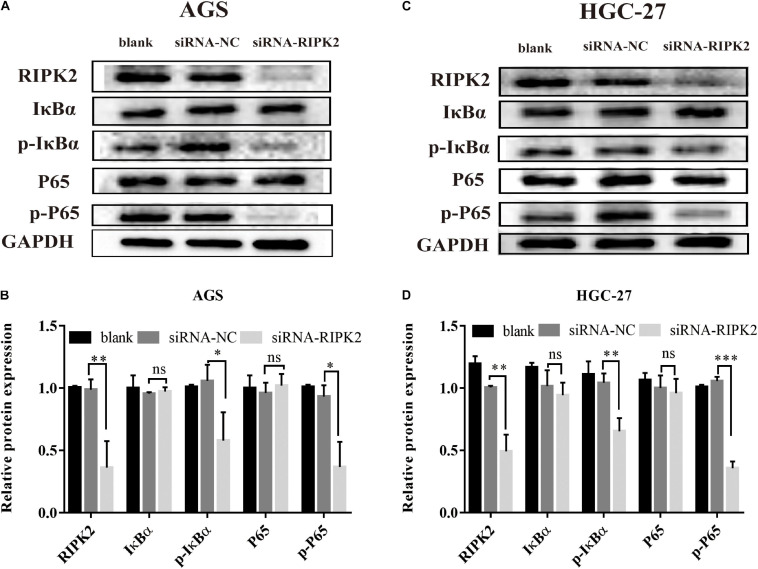
Knockdown of RIPK2 suppressed the NF-κB signaling pathway. **(A,B)** AGS cells and **(C,D)** HGC-27 cells were transfected with siRNA-NC or siRNA-RIPK2 and the protein levels of NF-κB (P65), phosphorylated P65, IκBα, and phosphorylated IκBα were measured by western blot (^∗^*P* < 0.05, ^∗∗^*P* < 0.01, ^∗∗∗^*P* < 0.001 versus siRNA-NC; two-tailed *t*-test). NC, negative control. ns, not significant.

The NF-κB pathway is critical for tumor formation and development. We predicted that the IκB-α/NF-κB pathway might play a role in the function of RIPK2 in GC (Figure 3). The western blotting analysis showed that the phosphorylation of P65 and IκB-α was decreased in the RIPK2-silenced AGS and HGC-27 cells (Figures 8A–D). These findings suggest that the observed regulation of proliferation, apoptosis, and migration in GC cells in response to RIPK2 knockdown, could be mediated *via* the NF-κB pathway.

## Discussion

RIPK2 is a serine/threonine/tyrosine kinase with a carboxy-terminal caspase activation and recruitment domain (Zare et al., 2018), which has been identified as a oncogene participating in several process involved in the development of tumors. Although the role of RIPK2 in cancer development has been investigated, especially in colorectal cancer (Chen et al., 2020), its function in GC is not yet clear. Only one study has suggested that the carriers of RIPK2 single nucleotide polymorphism rs16900627 minor allele may have a higher risk for the progression of gastric cancer (Ota et al., 2018).

A major goal of cancer research is to identify the alterations driving tumorigenesis (Hu et al., 2018). In our study, first, we explored RIPK2 expression levels in cancers based on the oncomine and TCGA databases. As expected, RIPK2 was overexpressed in various types of cancer tissues compared with corresponding normal tissues. Next, we evaluated the GC microarray data from three independent online public databases, and found that both RIPK2 mRNA and protein expression levels were significantly upregulated in GC tissues compared to that in normal tissues. Combined with the ROC analysis, our findings suggest that RIPK2 expression levels have the potential to become a novel diagnostic biomarker for GC.

To elucidate the role of RIPK2 in GC, we investigated the RIPK2-associated proteins using PPI network analysis. We found that the genes identified during this analysis were implicated in the modulation of apoptosis, immune response and cytokine production and, therefore, could be contributing to tumor (especially inflammatory carcinoma) formation and development. For example, NOD-like receptors are involved in innate immunity and the formation of inflammasomes, and can activate NF-κB, inflammatory caspases, and autophagy (Velloso et al., 2019). NOD1 and NOD2 are sensors of different bacterial peptidoglycan components. It has been previously shown that the activation of NOD1 promotes colon cancer metastases (Jiang et al., 2020); inhibition of RIPK2 can delay NOD signaling to decrease the production of inflammatory cytokine (Nachbur et al., 2015), so RIPK2 could regulate the NOD signaling to control the occurrence and development of inflammation-related tumors. Collectively, these genes were predicted based on the neighborhood, co-expression, database, experiment data, co-occurrence, and text mining. Further work is required to provide insight into the association of each gene with respect to RIPK2 in GC.

RIPK2 has been shown to affect cell growth and, therefore, may have a significant effect on cell viability and biological function (McCarthy et al., 1998). Two important study demonstrated that *Fusobacterium nucleatum*, a well-recognized proinflammatory bacterium, can facilitate ulcerative colitis and promote colorectal cancer metastasis *via* upregulation of RIPK2 expression (Chen et al., 2019, 2020). In addition, increased RIPK2 activity contributing to inflammatory breast cancer pathogenesis and aggressiveness (Zare et al., 2018). Thus, we hypothesize that RIPK2 might also serve as a biomarker for GC progression. With regard to the current study, to explore the role of RIPK2 in GC cells, HGC-27 and AGS cells were selected to perform loss-of-function experiments. Our results demonstrated that silencing of RIPK2 significantly decreased the proliferative capacity of AGS and HGC-27 cells. These findings are consistent with previous results showing that RIPK2 may play a vital role in the cancer cell growth (Wu et al., 2012; Zhang and Chin, 2014).

Apoptosis and proliferation are two important cellular processes involved in the development of cancer (Fesik, 2005). Our results demonstrated that silencing RIPK2 significantly increased the apoptosis rate in both AGS and HGC-27 cells. Moreover, we found that knockdown of RIPK2 induced apoptosis was related to regulate the Bcl2 family expression. The Bcl2 protein family is a large family of apoptosis modulating proteins that regulate the mitochondrial-mediated intrinsic pathway and includes anti-apoptotic proteins and pro-apoptotic proteins such as Bcl-2 and Bax (Delbridge et al., 2016). In our study, western blotting showed that the expression level of apoptotic protein Bax, as well as cleaved caspase-3, were increased, while anti-apoptotic protein Bcl2 expression was decreased. It was suggested that RIPK2 is responsible for the suppression of apoptosis in human GC.

Tumor cell migration is an important step and prerequisite for tumor invasion. Our previous research indicated that RIPK2 facilitated the colorectal cancer cells migration and invasion *via* inducing epithelial-mesenchymal transition (Chen et al., 2020). Thus, we performed transwell and wound healing assays to determine whether RIPK2 influenced the migration of GC cells. The results showed that silencing of RIPK2 significantly inhibited the AGS and HGC-27 cells migration which suggested that RIPK2 promoted cell migration behavior in GC cells.

It is well known that NF-κB plays an essential role in cell growth, differentiation, apoptosis, invasion, and metastases (Dolcet et al., 2005; Scholz and Taylor, 2013). The KEGG pathway analysis indicated that the IκB-α/NF-κB signaling pathway may function downstream of RIPK2 in GC. Since RIPK2 has sequence homology to RIP, a known activator of NF-κB (McCarthy et al., 1998), we investigated a possible role of RIPK2 in NF-κB activation in GC. In the present study, silencing of RIPK2 was found to suppress IκBα by phosphorylation at Ser32 sites, and the phosphorylation activity of NF-κB (P65) was positively regulated by RIPK2. Therefore, our study indicate that targeting RIPK2 could provide a potential strategy for GC therapy through deactivating IκBα/NF-κB signaling.

In summary, data from GEO, TCGA, and oncomine datasets, as well as comprehensive bioinformatics analyses, allowed us to identify RIPK2 as a candidate gene for GC diagnosis. Our results demonstrated that RIPK2 was significantly overexpressed in GC tissues. We also showed that RIPK2 was involved in cell proliferation, apoptosis, and migration, while knockdown of RIPK2 had a suppressive effect on tumorigenesis in GC cells, by downregulating the NF-κB activation.

However, our study has limitations: First, our cohort of patients with GC from our hospital was relatively small. Secondly, we only investigated silencing of RIPK2 *in vitro* studies. These aspects need to be addressed in future work.

In conclusion, this study demonstrated that RIPK2 was significantly upregulated in GC tissues. Furthermore, silencing of RIPK2 expression inhibited the GC cell growth and migration, induced apoptosis by suppressing the NF-κB signaling. Our results suggest that RIPK2 could be a novel therapeutic target for GC and provide new insights into the potential mechanism for the treatment of GC.

## Data Availability Statement

The original contributions presented in the study are included in the article/Supplementary Material, further inquiries can be directed to the corresponding author/s.

## Ethics Statement

The studies involving human participants were reviewed and approved by the Institutional Review Board of the Renmin Hospital of Wuhan University. The patients/participants provided their written informed consent to participate in this study.

## Author Contributions

QY was responsible for the conception and design of bioinformatic analysis, experiments, and original draft preparation. ST was responsible for data collection and statistical analysis. ZL was responsible for data curation. WD was responsible for providing experimental funds and technical guidance. All authors have read and approved the final manuscript.

## Conflict of Interest

The authors declare that the research was conducted in the absence of any commercial or financial relationships that could be construed as a potential conflict of interest.

## Footnotes

1 https://www.oncomine.org/

2 https://cistrome.shinyapps.io/timer

3 https://portal.gdc.cancer.gov/

4 http://www.ncbi.nlm.nih.gov/geo

5 https://www.proteinatlas.org/

6 http://string-db.org

7 http://david.ncifcrf.gov

8 http://www.sangerbox.com/tool

## Abbreviations

GC: gastric cancer
DEGs: differentially expressed genes
GO: gene ontology
BP: biological process
CC: cellular component
MF: molecular function
KEGG: Kyoto encyclopedia of genes and genomes
PPI: protein-protein interaction
STRING: search tool for the retrieval of interacting genes
TCGA: The cancer genome atlas
TIMER: tumor immune estimation resource
ROC: receiver operating characteristic
AUC: area under the ROC curve
HPA: human protein atlas
AV: annexin V FITC
PI: propidium iodide.
