# Supplementary material for: Knockdown of RIPK2 Inhibits Proliferation and Migration, and Induces Apoptosis via the NF-κB Signaling Pathway in Gastric Cancer
Source: Front Genet. 2021 Feb 9;12:627464. doi: 10.3389/fgene.2021.627464 (PMC7900563; doi:10.3389/fgene.2021.627464)
Supplement: Supplementary file 1 [file Data_Sheet_1.PDF]

Supplementary Table 1 RIPK2 expression in multiple cancers in Oncomine

ID-Name	Dataset	Cancer /tumor subtype	N	Reporter	t-Test	p	Fold change
RIPK2	Richardson Breast 2	Ductal Breast Carcinoma	40	209544_at	9.254	4.23E-12	2.381
RIPK2	Curtis Breast	Medullary Breast Carcinoma	32	LIMN_1758939	8.371	5.66E-10	2.309
RIPK2	Gaedcke Colorectal	Rectal Adenocarcinoma	65	A_23_P252106	22.269	2.81E-42	3.05
RIPK2	TCGA Colorectal	Colon Adenocarcinoma	101	A_23_P252106	21.162	7.32E-32	2.807
RIPK2		Cecum Adenocarcinoma	22	A_23_P252106	14.64	3.23E-16	2.871
RIPK2		Rectal Adenocarcinoma	60	A_23_P252106	15.656	5.94E-26	2.519
RIPK2		Colon Mucinous Adenocarcinoma	22	A_23_P252106	13.546	7.94E-15	2.876
RIPK2		Rectal Mucinous Adenocarcinoma	6	A_23_P252106	8.93	2.88E-07	2.415
RIPK2	Sabates-Bellver Colon	Colon Adenoma	25	209545_s_at	9.951	8.62E-14	2.428
RIPK2		Rectal Adenoma	7	209545_s_at	5.333	0.0000476	3.046
RIPK2	Hong Colorectal	Colorectal Carcinoma	70	209544__at	12.644	3.92E-10	4.069
RIPK2	Skrzypczak Colorectal	Colorectal Carcinoma	36	209544__at	9.413	1.6E-13	1.667
RIPK2		Colorectal Adenocarcinoma	45	209544__at	6.632	8.11E-09	1.317
RIPK2		Colon Carcinoma	5	209544__at	19.994	6.4E-08	3.307
RIPK2		Colon Adenoma	5	209544__at	12.543	0.00000373	2.262
RIPK2	Hao Esophagus	Esophageal Adenocarcinoma	5	IMAGE:1525461	7.108	0.00000341	3.515
RIPK2	DErrico Gastric	Gastric Mixed Adenocarcinoma	4	209544__at	7.026	6.99E-07	3.151
RIPK2		Gastric Intestinal Type Adenocarcinoma	26	209544__at	6.389	2.26E-08	2.824
RIPK2	Ginos Head-Neck	Head and Neck Squamous Cell Carcinoma	41	209545_s_at	8.568	1.8E-09	2.361
RIPK2	Peng Head-Neck	Oral Cavity Squamous Cell Carcinoma	57	3106243	9.467	1.24E-12	2.496

RIPK2	FriersonHF Salivary-gland	Salivary Gland Adenoid Cystic Carcinoma	16	41384_at	-4.579	0.000091	-7.796
RIPK2	Yusenko Renal	Clear Cell Renal Cell Carcinoma	26	209545_s_at	10.079	1.59E-10	10.079
RIPK2		Papillary Renal Cell Carcinoma	19	209545_s_at	6.034	0.00000261	2.818
RIPK2	Beroukhim Renal	Non-Hereditary Clear Cell Renal Cell Carcinoma	27	209545_s_at	7.463	2.44E-08	2.183
RIPK2		Hereditary Clear Cell Renal Cell Carcinoma	32	209544__at	8.519	1.25E-08	2.238
RIPK2	Jones Renal	Renal Oncocytoma	12	209544__at	-27.369	4.3E-18	-4.664
RIPK2		Renal Pelvis Urothelial Carcinoma	8	209544__at	-16.345	1.83E-08	-3.973
RIPK2	Coustan-Smith Leukemia	T-Cell Childhood Acute Lymphoblastic Leukemia	46	209545_s_at	-8.425	9.74E-09	-3.302
RIPK2	Haferlach Leukemia	T-Cell Acute Lymphoblastic Leukemia	174	209545_s_at	-12.359	2.26E-26	-3.269
RIPK2	Wurmbach Liver	Hepatocellular Carcinoma	35	209545_s_at	4.435	0.0000487	2.025
RIPK2	Zhan Myeloma 3	Smoldering Myeloma	12	209544__at	6.282	0.00000145	2.119
RIPK2	Detwiller Sarcoma	Malignant Fibrous Histiocytoma	9	209545_s_at	7.051	2.86E-07	3.032
RIPK2		Pleomorphic Liposarcoma	3	209545_s_at	5.414	0.0000463	2.329
RIPK2	Pei Pancreas	Pancreatic Carcinoma	36	209545_s_at	4.87	0.0000379	2.656

Note: P <0.05 was considered statistically significant
